# Molecular Dynamics Study of the Binding of Cationic, Anionic, and Neutral Luminescent Conjugated Ligands to the Alzheimer Folds of A*β*(1–42) and Tau Fibrils

**DOI:** 10.1002/cbic.202500902

**Published:** 2026-04-24

**Authors:** Yogesh Todarwal, Mathieu Linares, Patrick Norman

**Affiliations:** ^1^ Division of Theoretical Chemistry and Biology School of Engineering Sciences in Chemistry Biotechnology and Health KTH Royal Institute of Technology Stockholm Sweden; ^2^ PDC Center for High Performance Computing School of Electrical Engineering and Computer Sciences KTH Royal Institute of Technology Stockholm Sweden

**Keywords:** A*β*(1–42), amyloid fibrils, luminescent‐conjugated oligothiophenes, molecular dynamics simulations, tau protein

## Abstract

Unbiased atomistic molecular dynamics simulations have been employed to examine the binding interactions between amyloid fibrils (A*β*(1–42) and tau) with various ligands: anionic pFTAA, qFTAA‐CN, neutral HS‐276, and cationic bTVBT4. The ligand–fibril interactions were analyzed in a two‐step approach. First, an analysis of the spatial distributions of the ligands around the amyloid fibril protofilaments was carried out to identify prospective binding sites. Second, the associated ligand–fibril binding energies at these sites were determined using umbrella sampling. The results reveal that pFTAA and qFTAA‐CN share common binding sites in both A*β*(1–42) and tau fibrils. Ligands bTVBT4 and HS‐276, on the other hand, are found to have different binding sites in A*β*(1–42) but share the same site in tau fibrils. An analysis of the spatial distributions of all ligand–fibril pair combinations under comparable conditions shows the lowest densities when bTVBT4 interacts with A*β*(1–42) and HS‐276 with the tau fibril. These findings are corroborated by fluorescence costaining experiments, in which bTVBT4 and HS‐276 show no correlation with A*β*‐specific and tau‐specific antibody markers, respectively. Further structural analysis reveals significant changes in the conformations of all ligands upon binding to the proteins compared to their conformations in aqueous solution. The binding of the anionic ligands (multiple localized negative charges) to fibrils is primarily driven by Coulombic forces, whereas the binding of the neutral HS‐276 and cationic bTVBT4 (single delocalized positive charge) is governed by Lennard–Jones interactions. This highlights the influence of charges on binding, providing insights for the design of future ligands targeting A*β*(1–42) and tau fibrils.

## Graphical abstract

Atomistic simulations reveal selective binding of luminescent ligands to amyloid fibrils. Highly charged anionic ligands, pFTAA and qFTAA‐CN, bind strongly to A*β*(1–42) and tau through Coulombic interactions, whereas neutral HS‐276 and cationic bTVBT4 show site‐dependent, weaker, or absent binding dominated by Lennard‐Jones interactions.

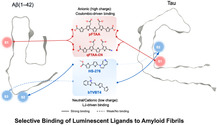

## 1 Introduction

Neurodegenerative diseases, such as Alzheimer's, Parkinson's, and Pick's, are associated with the aggregation of misfolded proteins where normally structured proteins lose their typical shape and form aggregates [1, 2, 3]. Particularly in Alzheimer's disease (AD), the misfolded proteins amyloid‐beta (A*β*(1–42)) and tau accumulate as deposits in the brain [4]. The pathogenesis of AD involves the development of A*β*(1–42) fibrils in the neocortex, which then spread into deeper brain regions [5, 6]. In contrast, tau fibril deposits originate in the limbic region and gradually extend outward toward the neocortex [7, 8, 9]. Recent literature suggests that such propagation is associated with a transition from the preclinical to the clinical phase [10, 11]. Therefore, the early detection of A*β*(1–42) and tau is of paramount importance, as it could potentially pave the way for timely interventions that might slow down the course of the disease.

One promising approach for detecting these pathological lesions involves the use of fluorescent ligands, which are molecules capable of binding to AD‐related lesions and emitting detectable light in the visible region of the electromagnetic spectrum. These ligands can be further converted into positron emission tomography tracers by labeling one of their atoms with a radioactive isotope, thereby enabling real‐time monitoring of AD pathogenesis [12]. The selection of appropriate fluorescent ligands is critical, as these ligands must possess specific characteristics to effectively detect AD‐related lesions [13]. First and foremost, these ligands should have the ability to penetrate the blood–brain barrier to ensure they can reach the target areas within the brain. Additionally, they should exhibit environmental sensitivity, meaning their optophysical properties change in response to different environments and result in spectral changes. These spectral changes can be attributed to both molecular and electronic structural alterations.

When these fluorescent ligands are adsorbed onto amyloid fibrils, the constrained environment typically triggers substantial conformational changes, which can enhance their fluorescence by adopting a more planar conformation that reduces nonradiative decay pathways. These conformational changes show the importance of considering both the physical constraints imposed by the binding site and the electronic effects exerted by surrounding residues, thereby shifting spectral properties. These electronic effects can be rigorously analyzed using quantum mechanics/molecular mechanics (QM/MM) simulations, as demonstrated in our previous studies [14, 15]. However, the current article will not address these electronic structural changes but will focus on the broader molecular dynamics (MD) simulations to explore the conformational and binding characteristics of the ligands.

To elucidate the molecular structural changes that underlie the desired environmental sensitivity and spectral shifts, MD simulations play a crucial role. By simulating the interactions between ligands and amyloid fibrils in different environments, such as water or within proteins, and calculating the dihedral distributions of important rotatable bonds, insights into the structural changes in varying environments can be gained. Furthermore, MD simulations can uncover the binding modes of ligands to protein fibrils and the associated energy landscapes. Comparative studies among multiple ligands can provide insights into the factors driving fibril binding, enabling the design of ligands that selectively bind to chosen sites while avoiding others. Elucidating specific binding sites is advantageous, as it facilitates the development of ligands tailored to target desired regions while avoiding unintended interactions. For instance, a new generation of ligands could be designed to avoid the binding sites common to the U.S. Food and Drug Administration approved ligand [18F]AV‐1451, which has been shown to produce misleading signals due to off‐target binding [16].

**FIGURE 1:**
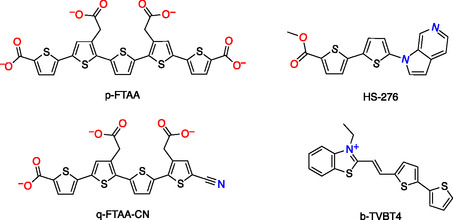
Molecular structures of the anionic ligands (pFTAA and qFTAA‐CN), neutral ligand (HS‐276), and cationic ligand (bTVBT4).

Ligands based on the thiophene moiety have shown promising results for binding to amyloid fibrils. Molecular structures of these ligands are shown in Figure 1. Combining the thiophene moiety with other structural motifs facilitates targeted protein binding. For example, the bTVBT4 ligand, which consists of a bithiophene unit combined with benzothiazole, binds exclusively to tau aggregates [17]. The HS‐276 ligand, comprising a bithiophene unit combined with an azaindole moiety, demonstrates preferential binding toward A*β*(1–42) aggregates [18]. The pFTAA, a luminescent conjugated polythiophene (LCP), binds to both hallmark tau and A*β*(1–42) folds of AD [19]. In contrast, qFTAA‐CN, another LCP ligand, binds to A*β*(1–42) [20], while its binding to tau aggregates remains ambiguous. Despite all four ligands sharing a thiophene moiety, they display diverse binding functionalities toward amyloids, making them excellent candidates for comparative computational studies.

In this study, we employ a computational approach to elucidate the differential binding affinities of pFTAA, qFTAA‐CN, HS‐276, and bTVBT4, when in complex with the proteins A*β*(1–42) and tau fibrils. These simulations are analyzed through a two‐step approach to provide a comprehensive comparative picture. First, we qualitatively assess the distribution of ligands around the protofilament of each fibril by examining their spatial distribution. Secondly, we perform a quantitative evaluation of the binding free energy. We also compare the structural characteristics of each ligand in the ligand–fibril complex to those in a water environment. The results suggest that anionic ligands carrying a high magnitude of charge bind through Coulombic interactions. In contrast, for neutral and cationic ligands with a single positive charge, it is driven by Lennard–Jones (LJ) interactions. Additionally, the characteristics of these ligands in the fibril complex significantly differ from their behavior in an aqueous environment by locking the ligand in a more planar conformation, enabling a stronger emission. This study therefore provides insights into the molecular mechanisms governing ligand–fibril interactions.

## 2 Computational Details

The binding interactions between A*β*(1–42) and tau fibrils with ligands pFTAA, qFTAA‐CN, HS‐276, and bTVBT4 were investigated using atomistic MD simulations. Comprehensive details of the computational methodology are provided in the supporting information (SI). The starting structures for A*β*(1–42) and tau fibrils were based on cryo‐EM structures and obtained from earlier research work [21, 22, 23]. The initial parameters for qFTAA‐CN, pFTAA, and HS‐276 ligands, sourced from the general amber force field [24, 25], were further refined and optimized using quantum chemical calculations. This refinement process included the use of the Gaussian program for obtaining optimized geometries and dihedral scanning [26, 27], and the Dalton program for performing single‐point absorption spectrum calculations [28], as elaborated in Section S1. The parameters for bTVBT4 were adopted from prior studies [22]. Amyloid fibrils consisting of amino acid residues were modeled using the standard amber force field parameters [29] and used the TIP3P force field for water molecules [30].

Section S2.2 elaborates on the MD simulations performed to identify potential binding sites on A*β*(1–42) and tau protein fibrils for each ligand. These calculations utilized the half‐pitch periodic model of either A*β*(1–42) or tau fibrils, combined with 60 ligand molecules, comprising 15 molecules of each ligand. The MD simulation trajectories were analyzed using the spatial distribution function tool developed by Skånberg et al*.* [31], within the VIAMD software to generate ligand density plots around the amyloid fibrils [32]. These plots were verified to have achieved convergence over the simulation time (see Figures S6 and S11 for details).

For a more in‐depth examination of the binding modes of each ligand at the identified sites, a smaller protein fibril model system was extracted from the half‐pitch periodic model, as described in Section S2.3. This reduced model comprises 10 filaments of either A*β*(1–42) or tau fibrils, each docked with one of the four ligands. To maintain the distance between amyloid filaments in the reduced model, position constraints were applied to the outermost protein filament during the MD simulations. From these smaller model simulations, strong binding modes for each ligand were identified through conformational analysis, selecting the most persistent and stable bound orientations that exhibited the highest occupancy throughout the simulation time. Starting from these identified strong binding modes, the free energy profile was determined using potential of mean force (PMF) calculations, employing the umbrella sampling method. A series of windows were generated along the reaction coordinate, defined as the distance between the center of mass of residues that form a binding site on a fibril and a ligand. Harmonic restraints were applied at each window to effectively sample the behavior of the system. Upon completing the MD simulations for each window, the weighted histogram analysis method was employed to compile the data and construct the PMF curve [33]. This curve reveals the free energy landscape of the ligand–fibril interaction. Further details about the procedure for obtaining the PMF profile and the parameters used are provided in Section S2.4.

Finally, simulations of pFTAA, qFTAA‐CN, and HS‐276 in a water solvent were also conducted to compare these results with those obtained in the protein environment. Details of these simulations are provided in Section S2.1. For bTVBT4 in water, the simulation data were sourced from previous work [22]. The planarity parameter used for the assessment of the molecular structure of ligands is adopted from Sjöqvist et al*.* [34] and given by

(1) $$P=\sum_{i=1}^{N}\frac{\left||\theta_{i}|-90\right|}{90}$$

where *θ* *_i_* are the dihedral angles in the molecule, and *N* is the number of dihedral angles for which the planarity is calculated. This parameter offers a quantitative measure of deviation from planarity, where higher values denote a more planar structure. When the planarity value approaches the total number of dihedral angles considered, the molecule is regarded as planar. Conversely, significant deviations from this value suggest a nonplanar structure.

## 3 Results and Discussion

In previous studies, unbiased MD simulations were used to identify the binding sites of anionic pentameric oligothiophene (pFTAA) and bithiophene‐benzothiazole (bTVBT4) when interacting with A*β*(1–42) and tau fibrils, respectively. In this work, we utilize a similar MD methodology to compare the binding properties of four ligands (pFTAA, qFTAA‐CN, HS‐276, and bTVBT4) to amyloid fibrils (A*β*(1–42) and tau). The structured cores of A*β*(1–42) and tau are potential targets for these ligands, as the disordered peptides attached to these cores are expected to dissociate [23, 35]. Therefore, we adopted the cryo‐EM structure‐based A*β*(1–42) and tau fibril models from our previous studies as the starting point for the simulations [21, 22]. The A*β*(1–42) and tau fibrils were simulated independently with an equal mixture of the four ligands (i.e., 15 molecules of each ligand type). To reduce any apparent cooperativity arising from multiple ligands, we used this elevated ligand concentration only to rapidly explore the protein–ligand energy landscape; however, for the spatial distribution analysis, only ligand molecules that bound to the fibrils as single units were considered. Through this analysis, the density of each ligand around the protein was comprehensively characterized, pinpointing the specific binding sites. By leveraging this visualization technique, we gained valuable insights into protein–ligand interactions and identified key residues that contribute to the formation of stable complexes for further analysis.

The following sections examine ligand interactions with amyloid fibrils and compare their behavior with that in an aqueous environment. First, we analyze the spatial distribution of ligands on half‐pitch models of A*β*(1–42) and tau fibrils to identify binding sites. Next, the driving forces behind ligand binding are explored through LJ and Coulombic interaction energies. The second section uses a small protein–ligand model to compare conformational analysis and planarity scores, highlighting differences between protein and aqueous environments that affect binding stability and interaction dynamics. The third section discusses ligand binding affinities based on PMF profiles, providing insights into the strength of ligand–fibril interaction. Finally, we reflect on the general binding patterns of these ligands.

### 3.1 Binding Sites

**FIGURE 2:**
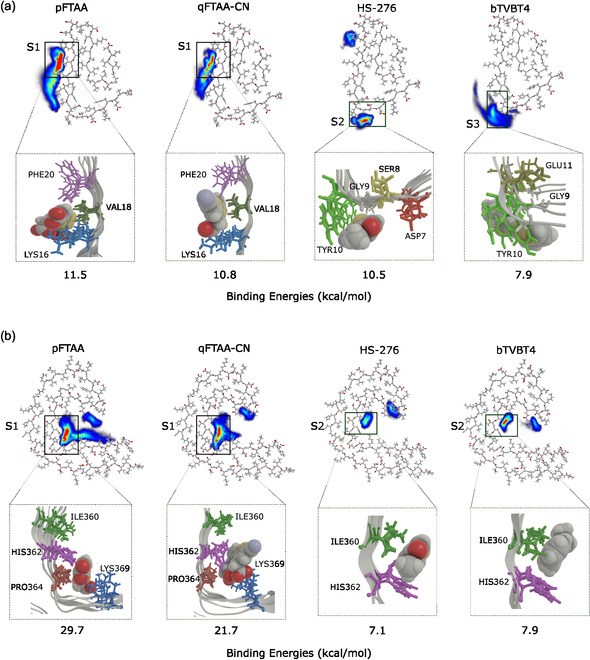
The spatial distribution of ligands around (a) A*β*(1–42) (parameters: density scaling = 9.5 and alpha scaling = 1.0) and (b) tau fibrils (parameters: density scaling = 5.0 and alpha scaling = 1.0). The plots are based on the last 100 ns of simulation data for systems containing 60 ligands (15 molecules of each ligand type) and either A*β*(1–42) or tau fibrils. Only ligands that were bound as monomers were considered.

The spatial distribution analysis reveals distinct binding sites for each ligand type (anionic, cationic, or neutral) on the A*β*(1–42) fibril (see Figure 2a). Interestingly, the anionic ligands pFTAA and qFTAA‐CN share the same primary binding site S1, which is located around amino acid residues LYS16, VAL18, and PHE20. The presence of the positively charged LYS16 likely attracts these negatively charged anionic ligands, contributing to the formation of a stable binding interaction. Not all charged residues constitute binding hot spots in the fibril, because many are buried or engaged in interstrand/protofilament contacts; persistent binding instead requires a solvent‐accessible charged “anchor” together with a complementary surface groove that matches the ligand shape and provides nearby hydrophobic or aromatic residues for van der Waals/*π*‐stacking stabilization. The neutral ligand HS‐276 binds to two sites on the A*β*(1–42) fibril, with the primary binding site S2 involving residues ASP7, SER8, GLY9, and TYR10, which form a pocket where HS‐276 appears to fit perfectly. However, at this site S2, it mainly interacts with the TYR10 and GLY9 residues (see Figure S9). The secondary binding site for HS‐276 is near residues PHE20 and ALA21, overlapping with the binding site for anionic ligands. The cationic ligand bTVBT4 exhibits a distinct binding site S3, comprising residues TYR10, GLU11, and VAL12, located near site S2. At binding site S3, bTVBT4 primarily interacts with TYR10 through *π* *–π* stacking, while its interaction with the negatively charged GLU11 residue is relatively weak because atoms bearing partial negative charges are positioned away from the ligand. Since it primarily interacts with a single residue, TYR10, this may explain the observed weak binding. Additionally, prolonged simulations of the smaller bTVBT4–A*β*(1–42) model system suggest that this binding is transient, as bTVBT4 eventually dissociates from the site.

The spatial distribution plots in Figure 2b illustrate the interactions between ligands and the tau fibril, highlighting binding sites for pFTAA, qFTAA‐CN, HS‐276, and bTVBT4. At site S1, pFTAA and qFTAA‐CN demonstrate a high population density, predominantly interacting with the residues ILE360, HIS362, PRO364, GLY367, and LYS369. At this site, analogous to their binding with A*β*(1–42), these anionic ligands display a strong affinity for the positively charged LYS369. They also show weaker interactions at two other sites, as shown in Figure 2b. The positively charged bTVBT4 and the neutral HS‐276 exhibit high population density at site S2, interacting with the amino acid residues ILE360, THR361, and HIS362. The population density of bTVBT4 is slightly higher compared to HS‐276 at site S2. Interestingly, there is one minor binding site common to all four ligands (see Figure 2b), which has been reported in both experimental and theoretical studies, suggesting that it is easily accessible to most ligands [36, 37, 38].

The interaction energies of the ligands when bound to the smaller protein fibril models (detailed in Section S2.3) are shown in Table 1. For both amyloid fibrils, the binding of the neutral ligand HS‐276 and the monocationic ligand bTVBT4 primarily exhibit LJ interactions, indicating that shape compatibility and hydrophobic effects are key drivers. Conversely, the binding of the anionic ligands pFTAA and qFTAA‐CN, which possess large charges, is predominantly driven by strong Coulombic interactions. This suggests that electrostatic compatibility is the main factor determining their binding affinity.

**TABLE 1:** LJ and Coulombic interaction energies (in kcal/mol) between ligands and amyloid fibrils (A*β*(1–42) or tau). Data extracted from a 500 ns (except for the bTVBT4—A*β*) MD simulation of a small amyloid fibril binding model. For calculation details, see SI Section 2.3.

Fibril	Type	pFTAA	qFTAA‐CN	HS‐276	bTVBT4
A*β*(1–42)	LJ	−26 ± 4	−24 ± 4	−26 ± 2	−24 ± 4
	Coulomb	−213 ± 33	−159 ± 35	−5 ± 6	−16 ± 5
tau	LJ	−28 ± 5	−31 ± 4	−26 ± 2	−24 ± 2
	Coulomb	−194 ± 32	−184 ± 24	−2 ± 2	−7 ± 3

### 3.2 Binding Modes

**FIGURE 3:**
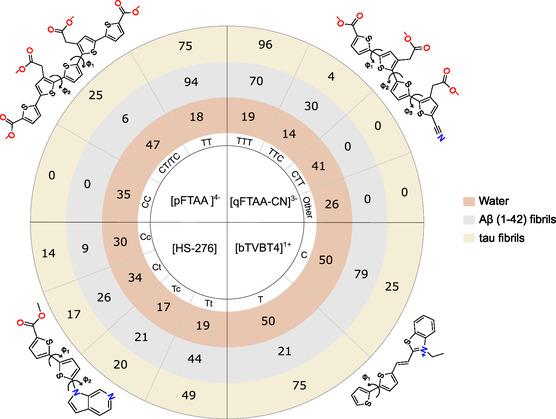
Conformers distributions of ligands with respect to the important dihedrals marked in the molecular structure illustrations in environments of water, A*β*(1–42), and tau. The dihedrals of type SCCS are marked with “T” or “C” and that of type SCCN is marked with “t” or “c.” The statistics for ligands in protein environments are determined from smaller binding models.

To further investigate the binding modes, smaller ligand–fibril models were used to reduce the computational complexity of the simulations. These models provided detailed insights into the conformational changes and the stability of ligand binding. The conformation distributions of ligands within the amyloid fibrils and aqueous environments exhibit substantial differences, which can be attributed to varying electrostatic and van der Waals interactions, among other factors. Importantly, these simulations revealed that ligands have greater planarity scores within protein environments than in water, enhancing their interaction efficiency with the protein surfaces. For clarity in the discussion that follows, uppercase letters (T or C) denote the SCCS type dihedrals, while lowercase letters (t or c) represent the SCCN type dihedral (see Figure 3).

**FIGURE 4:**
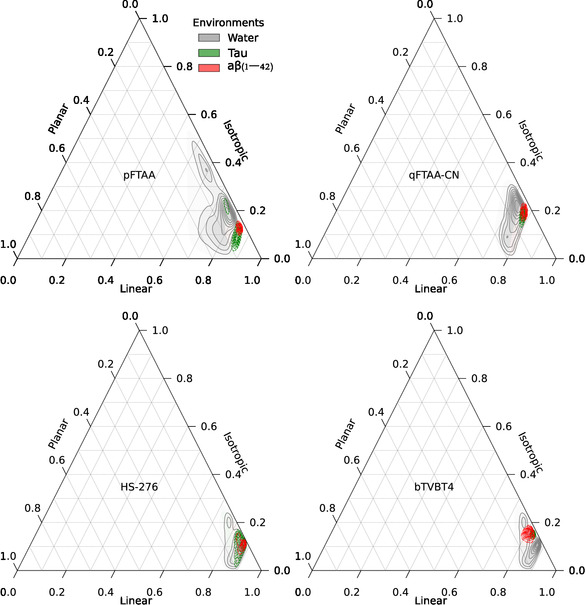
Ternary contour plots illustrate the conformational distributions of ligands pFTAA, qFTAA‐CN, HS‐276, and bTVBT4 in different environments: water (gray contours), tau (green contours), and A*β*(1–42) (red contours). For each simulation frame, three coordinates (linear, planar, and isotropic) were calculated using the VIAMD program, then normalized and projected onto ternary plots. The contours represent Gaussian kernel density estimations of these normalized data, highlighting regions of significant conformational density. The plots reveal how different environments influence the conformational behavior of each ligand.

#### 3.2.1 pFTAA and qFTAA‐CN

Within the major binding sites of A*β*(1–42) and tau fibrils, pFTAA predominantly adopts a TT conformation (94% in A*β*, 75% in tau), while qFTAA‐CN favors a TTT conformation (70% in A*β*, 96% in tau). In aqueous conditions, these ligands display multiple conformations, and the prevalence of these conformations drastically reduces to 18% for TT and 19% for TTT, indicating the impact of environmental constraints on ligand behavior. The planarity scores for both ligands are slightly higher in the protein environments (pFTAA: 2.86 in both A*β* and tau; qFTAA‐CN: 2.30 in A*β*, and 2.36 in tau) than in water (pFTAA: 2.77, qFTAA‐CN: 2.15), suggesting a more planar alignment (see Figure 4). These changes can be attributed to the structural alignment of their carboxyl groups in a specific orientation, facilitating stable protein–ligand complexes through strong Coulombic interactions with positively charged lysine residues (LYS16 in A*β* and LYS369 in tau), an essential factor for the binding efficacy of these anionic ligands.

#### 3.2.2 HS‐276 and bTVBT4

**FIGURE 5:**
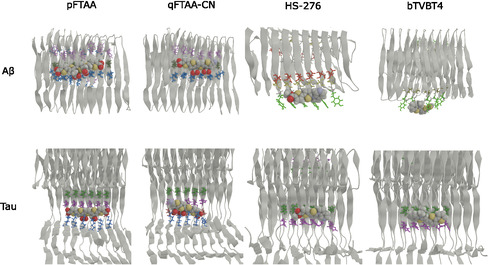
Top view of four ligands (pFTAA, qFTAA‐CN, HS‐276, and bTVBT4) interacting with A*β*(1–42) and tau fibrils. The important residues around the ligands are highlighted, and the names of these residues are shown in Figure 2.

HS‐276 exhibits a range of conformations in the major binding sites of both A*β* and tau fibrils, with the Tt conformation predominating in both types of amyloid fibrils. However, this distribution notably shifts toward a more diverse conformational spectrum under aqueous conditions, where the prevalence of the Tt conformation decreases from 49% in tau and 44% in A*β* to 19%. The planarity score for HS‐276 is slightly higher in protein environments (1.37 in A*β* and 1.36 in tau) compared to water (1.26), indicating a modest increase in planarity upon protein binding. The ligand bTVBT4 also shows variability, with an increased percentage of 75% in the T conformation when bound to tau and an increased percentage of 79% in the C conformation when bound to A*β*, compared to 50% for each in water. The planarity score for bTVBT4 increases in tau (3.46) and decreases in A*β* (3.35) compared to water (3.38). The lower planarity score in A*β* binding sites may be the reason leading to transient binding interactions with amyloid‐beta, as the reduced planarity might result in less stable interactions (Figure 5). Overall, these shifts in conformational patterns and planarity are mainly attributed to the dihedral type SCCS, which exhibits specific preferences in amyloids, suggesting that ligand binding induces structural alterations that could modify the photophysical properties of the ligands.

### 3.3 Binding Affinities

**FIGURE 6:**
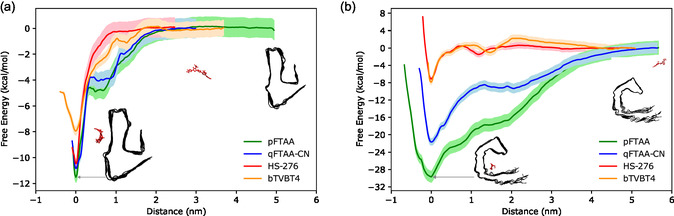
The PMF profiles obtained from multiple pulling trajectories for each of the four ligands when interacting with the (a) A*β*(1–42) and (b) tau fibrils, analyzed using the weighted histogram analysis method. The PMF profiles were generated by pulling the ligands from their most stable conformer at their respective major binding sites.

The free energy profiles of the ligands binding to the A*β*(1–42) are shown in Figure 6a. Both anionic ligands, pFTAA and qFTAA‐CN, exhibit similar profiles and start to interact with the fibril when they are within a distance of 2 nm from the binding site region. At ≈0.5 nm from this region, there is an intermediate state where only one carboxyl group of the ligand interacts with LYS16. The PMF profile for HS‐276 indicates that it starts to strongly interact with its binding site from ≈1.0 nm distance, while bTVBT4 starts interacting with its binding site at about 1.5 nm. The calculated binding energies from these PMF profiles reveal that pFTAA has the highest binding energy (11.5 kcal/mol), followed by qFTAA‐CN (10.8 kcal/mol) and HS‐276 (10.5 kcal/mol), while bTVBT4 displays the lowest binding energy (7.94 kcal/mol). These findings are consistent with previous experimental studies demonstrating the potential of pFTAA, qFTAA‐CN, and HS‐276 as reliable amyloid‐binding probes and confirming their binding to A*β*(1–42) fibrils [18, 19, 20]. The low binding energy of bTVBT4 aligns with fluorescence costaining experiments where bTVBT4 does not show correlation with A*β*‐specific antibody markers, suggesting that it may not bind to A*β*(1–42) fibril and might not be suitable for diagnosis compared to the other three ligands [17]. Interestingly, BTA‐3, a neutral ligand based on the benzothiazole moiety combined with two cyanide groups, has been reported to bind strongly at surface site S1 of A*β*(1–42) [39]. In contrast, bTVBT4, which also contains a benzothiazole moiety, shows decreased affinity. This suggests that the combination of benzothiazole with bithiophene in bTVBT4 reduces its binding to A*β*(1–42). Moreover, both BTA‐3 and qFTAA‐CN possess cyanide groups, which might be helping them to bind strongly at the same site S1.

The binding energies between ligands and tau fibrils provide further insights into the comparative strength of ligand–tau interactions. The PMF profiles show that pFTAA exhibits the highest binding energy of 29.7 kcal/mol at site S1, followed closely by qFTAA‐CN with a binding energy of 21.7 kcal/mol at the same site. At site S2, bTVBT4 has a binding energy of 7.9 kcal/mol, while HS‐276 shows a slightly lower binding energy of 7.1 kcal/mol. Moreover, the binding PMF profiles of pFTAA and qFTAA‐CN are remarkably similar, suggesting a barrierless binding process to site S1, with interactions commencing from a considerable distance. The ligands bTVBT4 and HS‐276 also exhibit comparable binding profiles and a barrierless path; however, their interaction with site S2 only initiates at a closer proximity to the binding site. Experimental results align with the calculated relative binding energies of the ligands, with pFTAA and bTVBT4 displaying fluorescence upon binding to tau fibrils, whereas HS‐276 having the lowest binding energy does not. However, the difference in binding energy between bTVBT4 (which fluoresces) and HS‐276 (which does not) is only 0.8 kcal/mol, falling within the error range of the PMF method used to calculate free energy. The ambiguous fluorescence behavior of qFTAA‐CN observed in some experiments might be attributed to posttranslational modifications of tau, specifically the acetylation of LYS369 at site S1, which could weaken its binding to tau and inhibit fluorescence [20, 40]. Further investigation is required to confirm this hypothesis and to explore whether pFTAA can bind to site S1 even after posttranslational modifications, especially given its higher binding energy compared to qFTAA‐CN.

The stronger binding of pFTAA and qFTAA‐CN to tau fibrils compared to A*β*(1–42) fibrils can be attributed to both structural and energetic factors. While A*β*'s site S1 provides close contacts with residues LYS16, VAL18, and PHE20, interactions begin at relatively shorter range (≈2 nm). In contrast, tau's site S1 exhibits a barrierless binding profile where ligand–fibril interactions commence from separations greater than 4 nm, as evident from the PMF profiles in Figure 6b. The groove‐like topology of tau's binding site, formed by a larger cluster of residues (ILE360, HIS362, PRO364, GLY367, and LYS369), creates a deeper binding pocket that accommodates the ligands more effectively. This extended residue cluster maintains persistent multiresidue contacts while allowing sufficient conformational flexibility, thereby optimizing both enthalpic contributions through stable interactions and entropic contributions through reduced conformational penalties. These combined factors result in the substantially deeper global minimum observed for tau binding (29.7 kcal/mol for pFTAA) compared to A*β* binding (11.5 kcal/mol).

The spatial distribution of ligands around both A*β*(1–42) and tau protofilaments, shown in Figures 2a,b, respectively, reveals significant variations in ligand population densities, which reflect their differential binding affinities. For A*β*(1–42), pFTAA, qFTAA‐CN, and HS‐276 exhibit higher population densities compared to bTVBT4, correlating with their stronger binding affinities toward the A*β* fibril. A similar pattern is observed around the tau protofilaments; here, HS‐276 shows a lower population density compared to the others, indicating a reduced affinity toward tau fibrils, while pFTAA, qFTAA‐CN, and bTVBT4 maintain high densities. These observations highlight how structural variations between ligands can impact their efficacy and specificity in binding to different types of amyloid fibrils, thus supporting the need for tailored approaches in the design and selection of diagnostic fluorescence ligands.

## 4 Conclusions

This study employed atomistic MD simulations to elucidate the binding interactions between four ligands (pFTAA, qFTAA‐CN, HS‐276, and bTVBT4) and amyloid fibrils (A*β*(1–42) and tau) associated with AD. Through a systematic analysis involving spatial distribution functions and binding free energy calculations, distinct binding sites and affinities were identified for each ligand on both amyloid fibrils. These results are supported by observations in experimental fluorescence spectra. The anionic ligands, pFTAA and qFTAA‐CN, were found to share a common binding site on both A*β*(1–42) and tau fibrils, with their binding primarily driven by favorable Coulombic interactions with the positively charged lysine residues. In contrast, the binding of the neutral HS‐276 and cationic bTVBT4 ligands is mainly governed by LJ interactions. Furthermore, the fibril environment induced significant conformational changes in the ligands compared to their behavior in aqueous environments, favoring more planar conformations that enhance fluorescence.

These mechanistic insights provide actionable guidance for the rational design of next‐generation diagnostic ligands. Specifically, the demonstrated importance of charge distribution (multiple localized charges for Coulombic‐driven binding versus neutral or monocationic structures for shape‐complementary interactions) and the identification of distinct binding sites for A*β*(1–42) versus tau fibrils enable structure‐guided optimization of ligand selectivity and affinity. This work thus establishes a computational framework for accelerating the development of improved molecular probes for early detection and monitoring of AD pathology.

## Supporting Information

Additional SI can be found online in the Supporting Information section.

## Funding

This work was supported by the Swedish Research Council (2023‐5171).

## Conflicts of Interest

The authors declare no conflicts of interest.

## Supporting information

Supplementary Material

## Data Availability

The data that support the findings of this study are available in the SI of this article.
