# Supplementary material for: Molecular Dynamics Study of the Binding of Cationic, Anionic, and Neutral Luminescent Conjugated Ligands to the Alzheimer Folds of Aβ(1–42) and Tau Fibrils
Source: Chembiochem. 2026 Apr 24;27(8):e202500902. doi: 10.1002/cbic.202500902 (PMC13109672; doi:10.1002/cbic.202500902)
Supplement: Supplementary file 1 — Supplementary Material [file CBIC-27-e202500902-s001.pdf]

Comparative Analysis of Fluorescent Cationic, Anionic, and Neutral Ligands to Amyloid Fibrils.

Yogesh Todarwal, Mathieu Linares, and Patrick Norman*

Division of Theoretical Chemistry and Biology, School of Engineering Sciences in Chemistry, Biotechnology and Health, KTH Royal Institute of Technology, SE-100 44 Stockholm, Sweden

E-mail: panor@kth.se

Supplementary Information

1 Molecular modeling of ligands pFTAA, qFTAA-CN, HS-276, and bTVBT4

1.1 Force field parametrization of qFTAA-CN

Conformation analysis

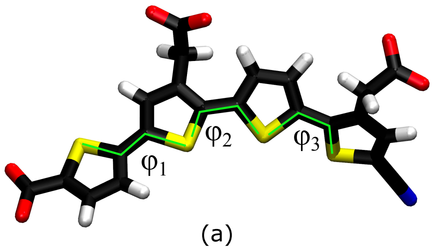

(a)

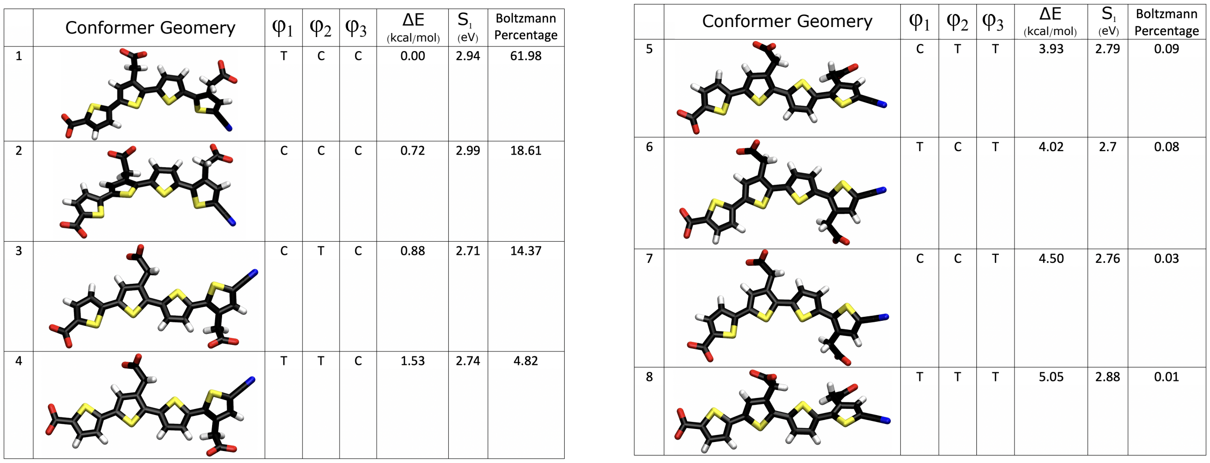

	Conformer Geomery	φ1	φ2	φ3	ΔE (kcal/mol)	S1 (eV)	Boltzmann Percentage
1		T	C	C	0.00	2.94	61.98
2		C	C	C	0.72	2.99	18.61
3		C	T	C	0.88	2.71	14.37
4		T	T	C	1.53	2.74	4.82
5		C	T	T	3.93	2.79	0.09
6		T	C	T	4.02	2.7	0.08
7		C	C	T	4.50	2.76	0.03
8		T	T	T	5.05	2.88	0.01

(b)

Figure S1: (a) Naming convention for important dihedral angles in qFTAA-CN. (b) Possible conformations of qFTAA-CN with relative ground state energy (ΔE) in kcal/mol, excitation energy (S_1) in eV, and Boltzmann percentage. All structures were optimized at the B3LYP/aug-cc-pVDZ, followed by frequency calculation at the same level. Subsequently, calculations of excitation energies were performed using the CAM-B3LYP/aug-cc-pVDZ level.

Validation of force field

Table S1: Comparison of the relative energies (in kcal/mol) of conformers: The geometries were optimized using both the B3LYP/aug-cc-pVDZ level of theory and the Molecular Mechanics (MM) method. The error denotes the absolute difference in the relative energies obtained from these two methods.

Conformer	ΔE_{DFT} (kcal/mol)	ΔE_{MM} (kcal/mol)	|Error| (kcal/mol)
TCC	0.0	0.0	0.0
CCC	0.7	0.4	0.3
CTC	0.9	-0.3	1.1
TTC	1.5	2.3	0.7
CTT	3.9	4.2	0.2
TCT	4.0	6.0	2.0
CCT	4.5	6.3	1.8
TTT	5.1	7.3	2.2

Table S2: Comparison of excitation energies (S_1) of conformers: calculated based on ground state geometries optimized using B3LYP (DFT) and the molecular mechanics (MM) method. The error represents the absolute difference in excitation energies between the two methods. The single-point excitation energies were calculated at the CAM-B3LYP/aug-cc-pVDZ level.

Conformer	S_1(DFT) (eV)	S_1(MM) (eV)	|Error| (eV)
TCC	2.94	2.94	0.00
CCC	2.99	3.04	0.05
CTC	2.71	2.84	0.13
TTC	2.74	2.83	0.09
CTT	2.79	2.89	0.10
TCT	2.70	2.90	0.20
CCT	2.76	2.97	0.21
TTT	2.88	2.94	0.06

1.2 Force field parametrization of pFTAA

Conformation analysis

	Conformer Geomery	φ1	φ2	ΔE (kcal/mol)	S_1 (eV)	Boltzmann Percentage
1	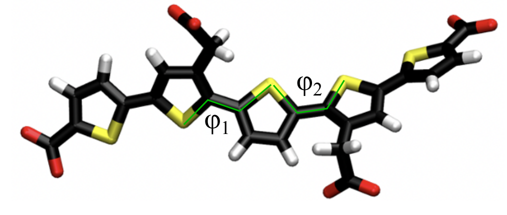	T	C	0.00	3.02	93.7
2	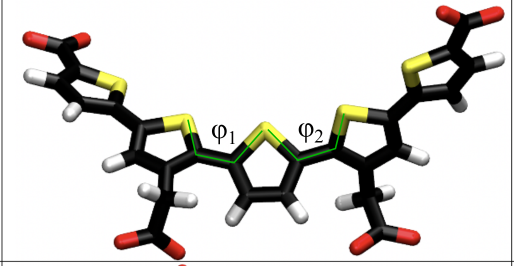	C	C	1.32	3.19	5.2
3	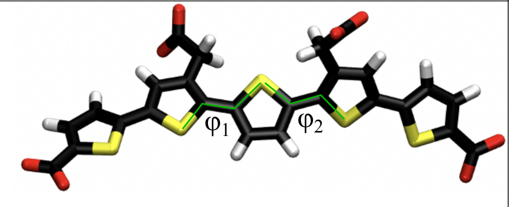	T	T	2.23	2.95	1.1

Figure S2: Conformational analysis of pFTAA: Displayed are the conformations characterized by two important inner dihedrals, detailing relative energies (ΔE) in kcal/mol, excitation energies (S_1) in eV, and their respective Boltzmann distributions. The structures were optimized using B3LYP/aug-cc-pVDZ. Excitation energies were subsequently determined through CAM-B3LYP/aug-cc-pVDZ level of theory.

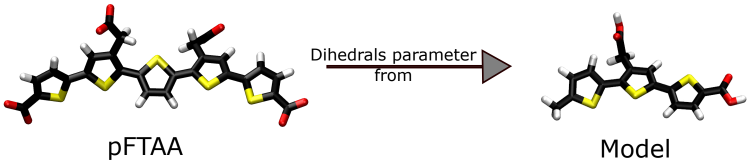

Figure S3: Molecular structure of the model from which force field parameters for pFTAA were derived.

Validation of force field

Table S3: Comparison of the relative energies (in kcal/mol) of conformers: The geometries were optimized using both the B3LYP/aug-cc-pVDZ level of theory and the Molecular Mechanics (MM) method. The error denotes the absolute difference in the relative energies obtained from these two methods.

Conformer	ΔE_{DFT} (kcal/mol)	ΔE_{MM} (kcal/mol)	$\|$Error$\|$ (kcal/mol)
TC	0.0	0.0	0.0
CC	1.3	-1.1	2.4
TT	2.2	2.6	0.4

Table S4: Comparison of excitation energies (S_1) of conformers: calculated on ground state geometries optimized in B3LYP (DFT) and molecular mechanics (MM) method. The error represents the absolute difference in excitation energies between the two methods. The single point excitation energies were calculated at CAM-B3LYP/aug-cc-pVDZ.

Conformer	S_1(DFT) (eV)	S_1(MM) (eV)	$\|$Error$\|$ (eV)
TC	3.02	3.01	0.01
CC	3.19	3.18	0.01
TT	2.95	2.97	0.02

1.3 Force field parametrization of HS-276

Conformation analysis

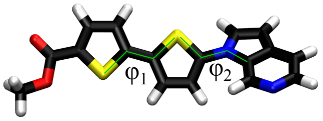

	Conformer Geomery	φ_1	φ_2	ΔE (kcal/mol)	S_1 (eV)	Boltzmann Percentage
1	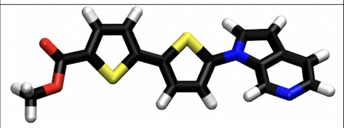	T	T	0.00	3.51	50.2
2	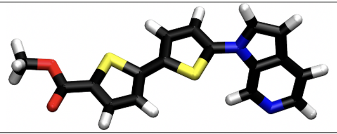	T	C	0.26	3.55	32.6
3	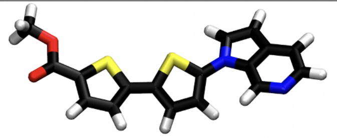	C	T	0.87	3.57	11.8
4	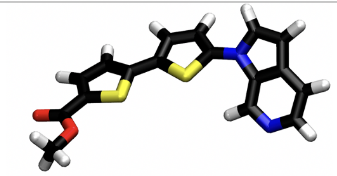	C	C	1.34	3.66	5.4

Figure S4: Conformational analysis of HS-276: Displayed are the conformations characterized by two rotatable dihedrals, detailing relative energies (ΔE) in kcal/mol, excitation energies (S_1) in eV, and their respective Boltzmann distributions. The structures were optimized using B3LYP/aug-cc-pVDZ. Excitation energies were subsequently determined through CAM-B3LYP/aug-cc-pVDZ level of theory.

Validation of force field

Table S5: Comparison of the relative energies (in kcal/mol) of conformers: The geometries were optimized using both the B3LYP/aug-cc-pVDZ level of theory and the Molecular Mechanics (MM) method. The error denotes the absolute difference in the relative energies obtained from these two methods.

Conformer	ΔE_{DFT} kcal/mol	ΔE_{MM} kcal/mol	|Error| (kcal/mol)
TT	0.0	0.0	0.0
TC	0.3	0.3	0.0
CT	0.9	1.2	0.3
CC	1.3	2.0	0.6

Table S6: Comparison of excitation energies (S_1 in eV) of conformers: calculated on ground state geometries optimized in B3LYP (DFT) and molecular mechanics (MM) method. The error represents the absolute difference in excitation energies between the two methods. The single point excitation energies were calculated at CAM-B3LYP/aug-cc-pVDZ.

Conformer	S_1(DFT) (eV)	S_1(MM) (eV)	|Error| (eV)
TT	3.51	3.49	0.02
TC	3.55	3.49	0.06
CT	3.57	3.60	0.03
CC	3.66	3.69	0.03

1.4 Force field parameters of bTVBT4

The force field parameters for bTVBT4 were adopted from previous studies.[1]

2 Molecular dynamics simulation

All atomistic MD simulations were performed using GROMACS (version 2022.2).[2–5] Force fields for the ligands pFTAA, qFTAA-CN, and HS-276 were reparameterized, starting from GAFF. We used an Amber ff14SB force field for amyloid fibrils,[6] TIP3P for water solvents,[7] and GAFF for counterions (Cl^- or Na^+). Before carrying out the MD simulation production run, all MD preprocessing steps such as energy minimization and equilibration were performed for all types of systems: each of the four ligands in a water solvent, periodic amyloid fibrils in water solvent with 60 ligands, or a ligand in a small amyloid fibril model. For all systems, the solvation box was neutralized with counter ions (Cl^- or Na^+). The protonation state of all amino acid residues in the amyloid fibrils is at physiological pH 7.4.

For the MD simulation in the NPT ensemble at the equilibration step, the velocity rescaling weak coupling scheme with a coupling constant of $\tau = 0.2$ ps was used to maintain the reference temperature of 300 K. The pressure was maintained at 1 atm using a Berendsen barostat with a coupling constant of 1 ps. The Berendsen barostat was used under semi-isotropic conditions for systems of amyloid fibrils with ligands. While for each ligand in water, the isotropic condition was applied. All three directions of the simulated box were subjected to periodic boundary conditions.

The particle mesh ewald (PME) approach[8] was used to model electrostatic interactions with a long-range cutoff of 1.5 nm. Lennard-Jones interactions were also modelled with the same cutoff of 1.5 nm. The time step for MD simulation was 2 fs, with a pair-list update period of 10 steps. The LINCS routine was used to keep all H-bond lengths constant.[9]

2.1 Molecular dynamics simulation of each ligand in water solvent

Starting from the B3LYP optimized structures, the ligands pFTAA, qFTAA-CN, and HS-276 were first solvated in water within an isotropic simulation box with side lengths of 6 nm, 5 nm, and 5 nm, respectively. Next, counter ions were added to neutralize the charge of

the systems. Each system was then energy minimized. This was followed by a short 100 ps simulation run in an NPT ensemble to stabilize the pressure while applying position restraint to the ligand. Upon completion of the equilibration phase, two independent MD simulations of 250 ns each, accumulating time a total time of 500 ns, were run in the NVT ensemble.

2.2 Modeling ligand interactions with a half-pitch periodic model of amyloid fibrils.

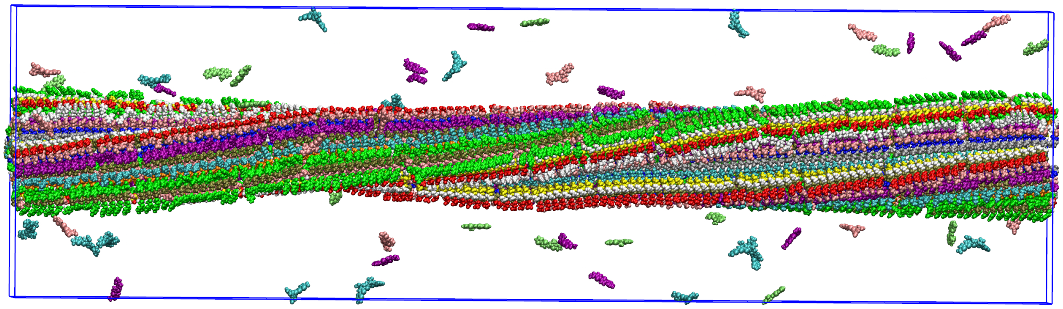

Figure S5: Illustration of a simulation box containing the half-pitch Aβ(1–42) fibril and 60 ligand molecules. Water and ions have been removed for clarity.

The starting structure of the half-pitch periodic model of amyloid fibrils (Aβ(1–42) and tau) for MD simulation was adopted from previous studies.[1,10] Next, the systems were built consisting of a protein fibril filled with 60 ligand molecules of four different types (comprising of 15 molecules of each of the four ligands). These 60 ligand molecules were added to the simulation box at random positions and orientations, which were then filled with water molecules and counter ions. The equilibration phase was performed by applying position restraints to an amyloid fibril and 60 ligand molecules. In the production phase of the MD simulation, each independent amyloid fibril was simulated for over 200 ns. Two additional systems were constructed for the tau fibril to increase sampling on its large surface: inside and outside the cavity. The results from MD simulations across various systems are depicted through spatial distribution plots. These plots are obtained by overlaying all protofilaments of the amyloid fibril on top of one another and then calculating the density of ligands around

the protofilaments over the simulation time.

Next, the spatial distribution plots exhibiting the interactions between various ligands and protein fibrils were thoroughly examined to find the strong binding sites. It is important to note that only ligand molecules that bound to the fibril as monomer units were considered for this analysis. The data were considered at three distinct time intervals: 50 ns, 100 ns, and 150 ns. The results for ligands interacting with both Aβ(1–42) and tau fibrils revealed that the most prominent binding site was identified as early as 50 ns. Interestingly, as time progressed to 100 ns and 150 ns, the binding site continued to refine, but the major information regarding the interaction remained consistent. This finding clearly demonstrates that the spatial distribution plots exhibit convergence, indicating the robustness of the identification of the strongest site.

Subsequently, in another analysis, the number of ligands at the identified binding sites was kept constant for each type, and the spatial distribution plots were recalculated (see Figure S7) to investigate the rigidity or dynamism of ligand binding at the interaction site. This provided a more in-depth understanding of the stability of the protein-ligand complex.

2.2.1 Results for Ligands Interacting with Aβ(1–42) Fibril

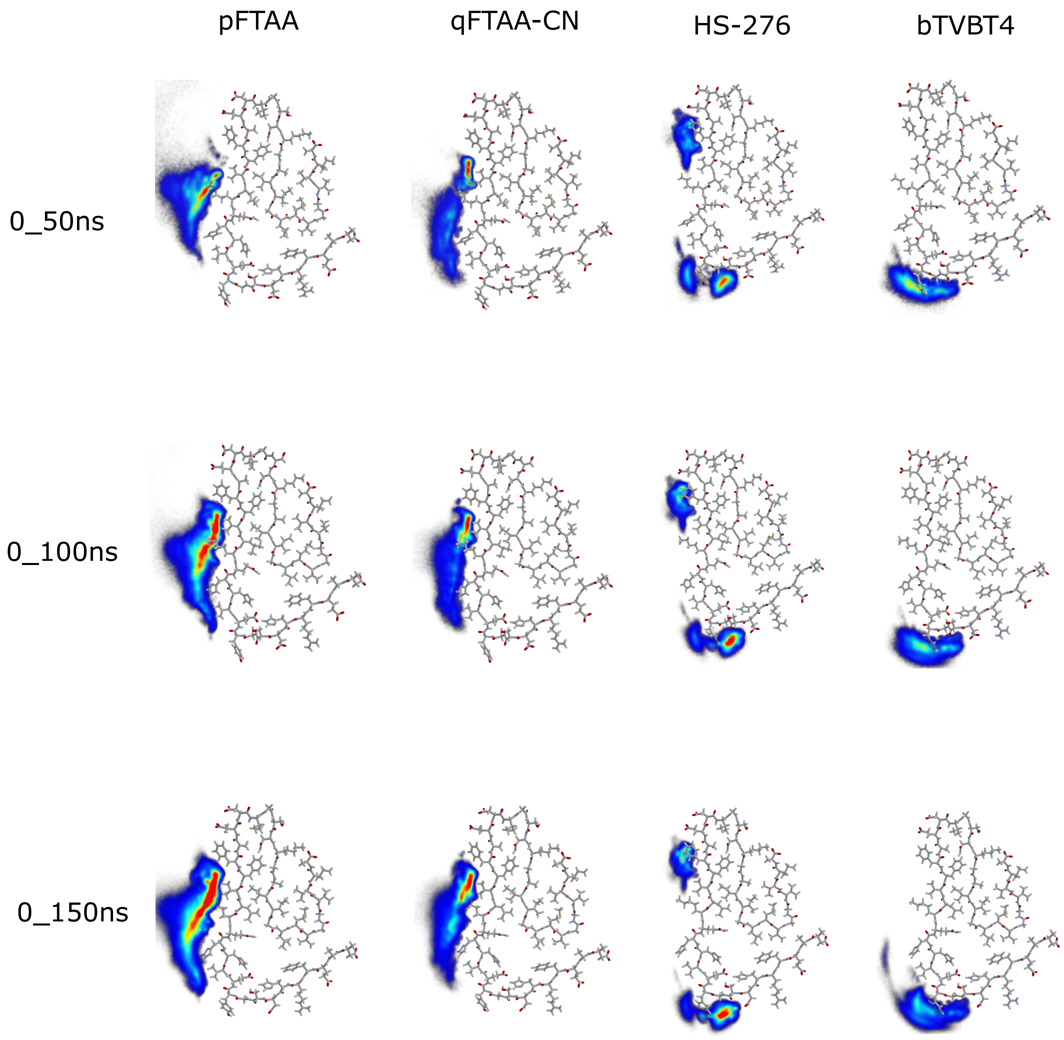

Figure S6: Convergence of spatial distribution plots for ligand molecules (15 each of pFTAA, qFTAA, HS276, and bTVBT4) interacting with half-pitch periodic Aβ(1–42) fibril model over 150 ns MD trajectory: (a) 0-50 ns, (b) 0-100 ns, (c) 0-150 ns. Plots converge after 100 ns. Only ligand molecules binding as monomers were considered for analysis.

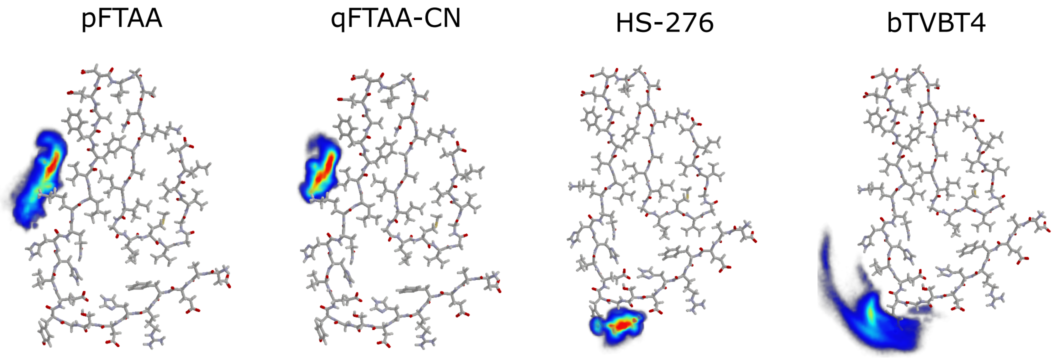

Figure S7: Spatial distribution plots for a constant number of pFTAA, qFTAA, HS276, and bTVBT4 ligands bound at their respective major binding sites on Aβ(1–42) fibrils, calculated over the 100-200 ns simulation trajectory.

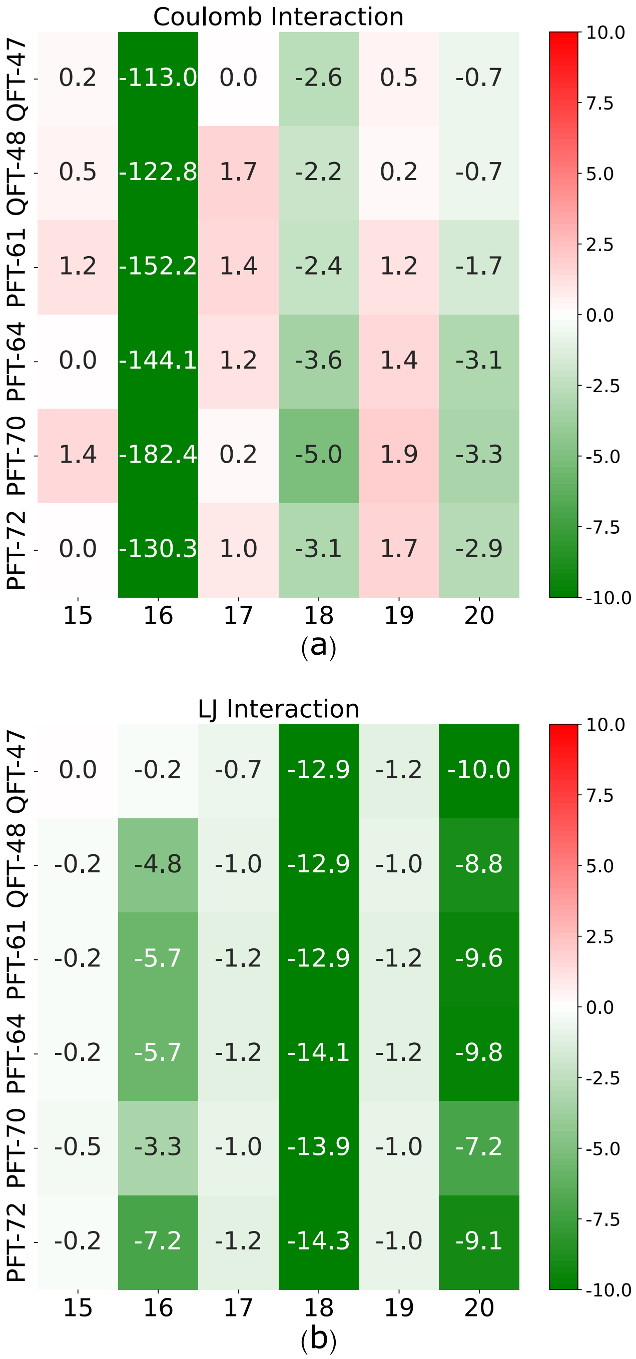

Figure S8: Interaction energies (in kcal/mol) between Aβ(1–42) peptide residues (GLN15, LYS16, LEU17, VAL18, PHE19, PHE20) and ligands pFTAA (marked as PFT) and qFTAA-CN (marked as QFT): (a) Coulombic interactions and (b) Lennard-Jones interactions.

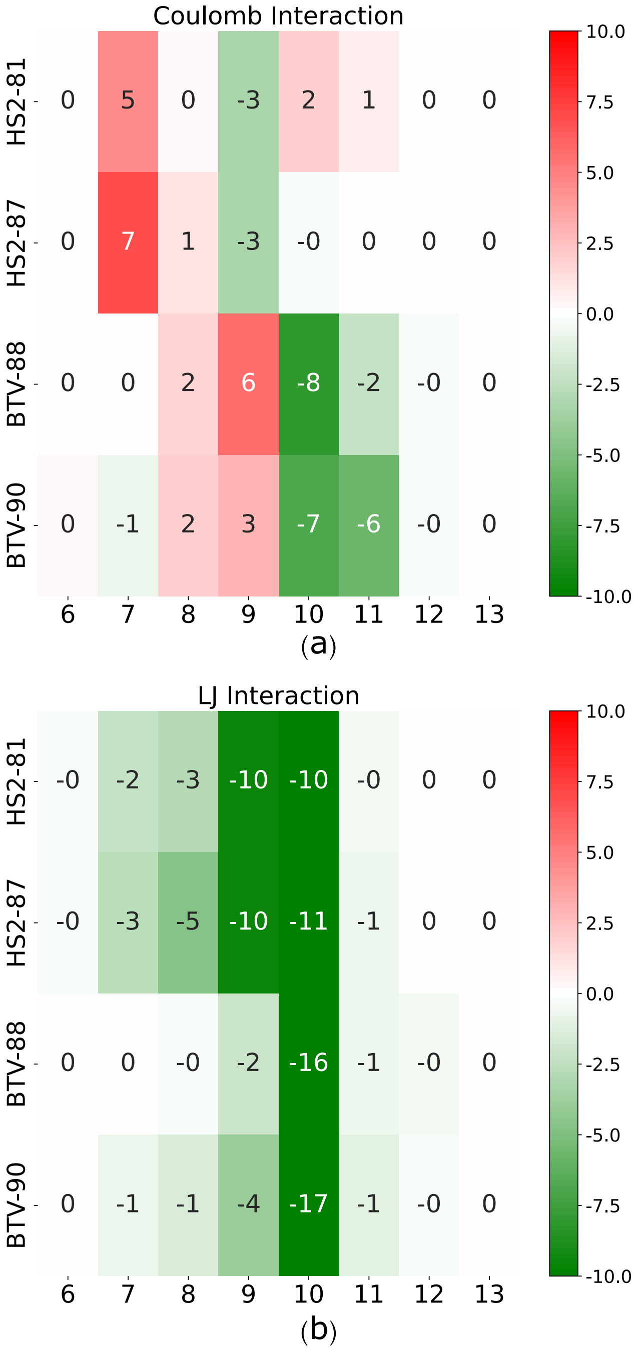

Figure S9: Interaction energies (in kcal/mol) between Aβ(1–42) peptide residues (HIS6, ASP7, SER8, GLY9, TYR10, GLU11, VAL12, HIS13) and ligands bTVBT4 (marked as BTV) and HS-276 (marked as HS2): (a) Coulombic interactions and (b) Lennard-Jones interactions.

2.2.2 Results for Ligands Interacting with Tau Fibril

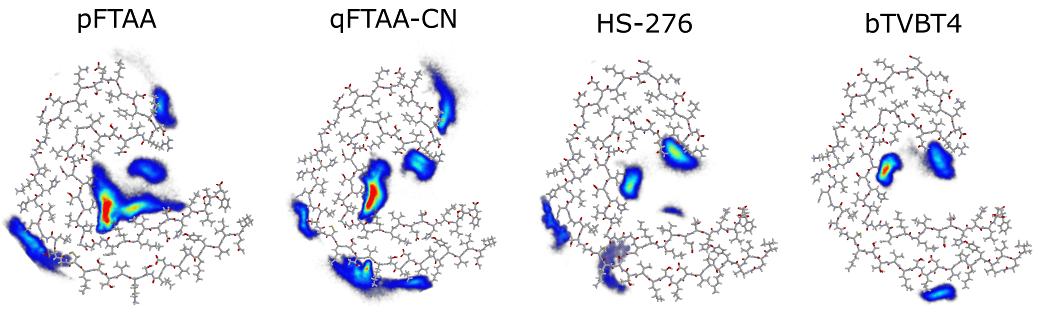

Figure S10: Ligand spatial density comparison within and outside the tau fibril cavity obtained from the MD simulation. For all the ligands, the density is higher inside than outside the cavity.

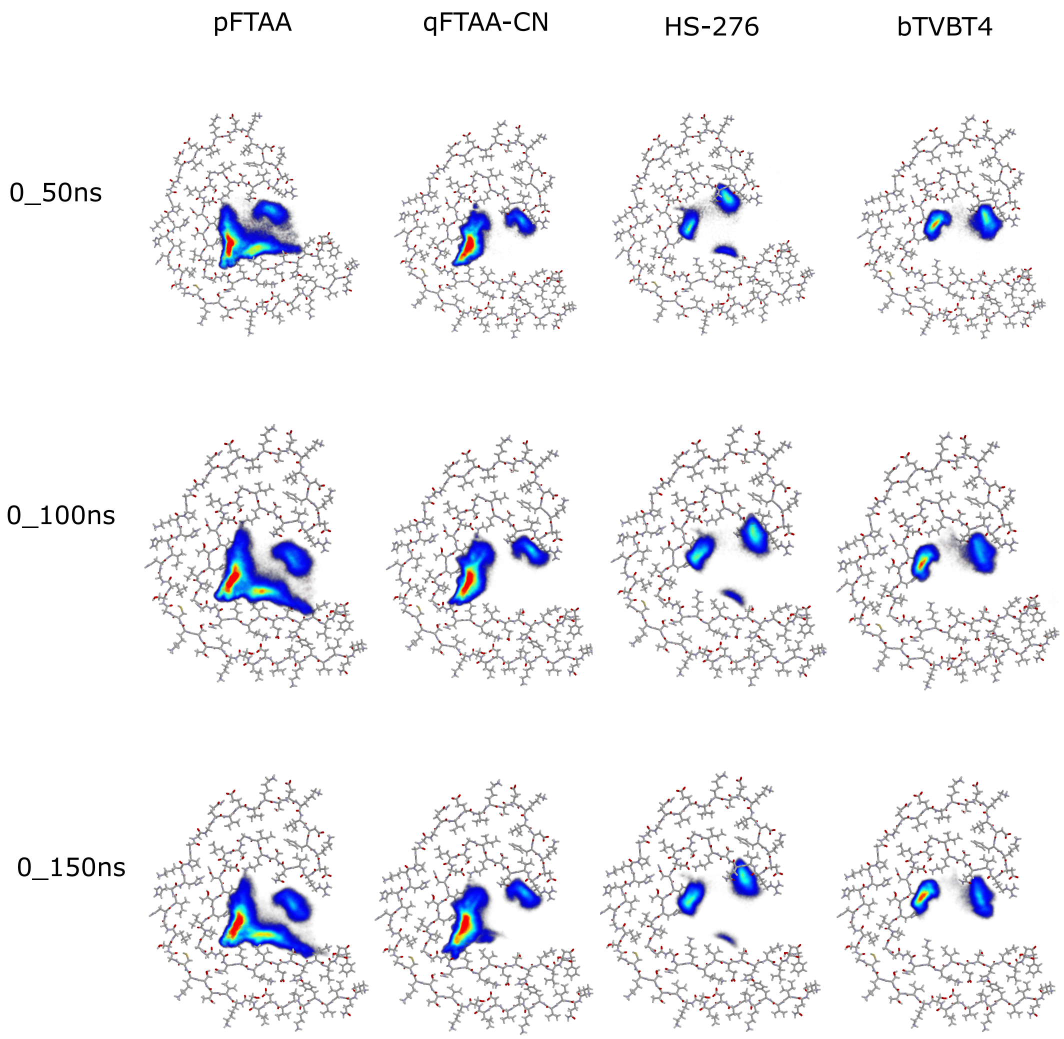

Figure S11: Convergence of spatial distribution plots for ligand molecules (15 each of pFTAA, qFTAA, HS276, and bTVBT4) interacting with half-pitch periodic tau fibril model over 150 ns MD trajectory: (a) 0-50 ns, (b) 0-100 ns, (c) 0-150 ns. Plots converge after 50 ns. Only ligand molecules binding as monomers were considered for analysis.

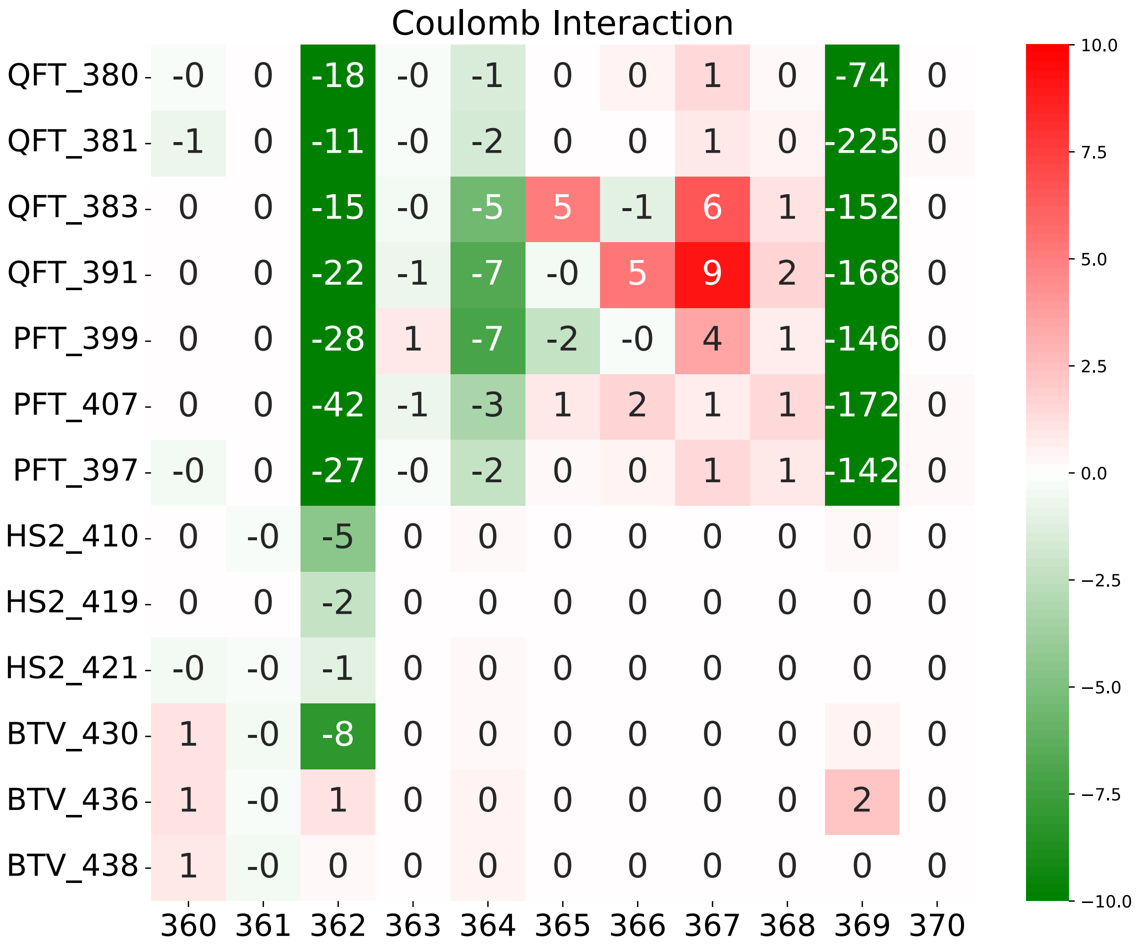

Figure S12: Coulombic interaction energies (in kcal/mol) between tau peptide residues ILE360, THR361, HIS362, VAL363, PRO364, GLY365, GLY366, GLY367, ASN368, LYS369, LYS370, and the ligands pFTAA (PFT), qFTAA (QFT), HS276 (HS2), and bTVBT4 (BTV)

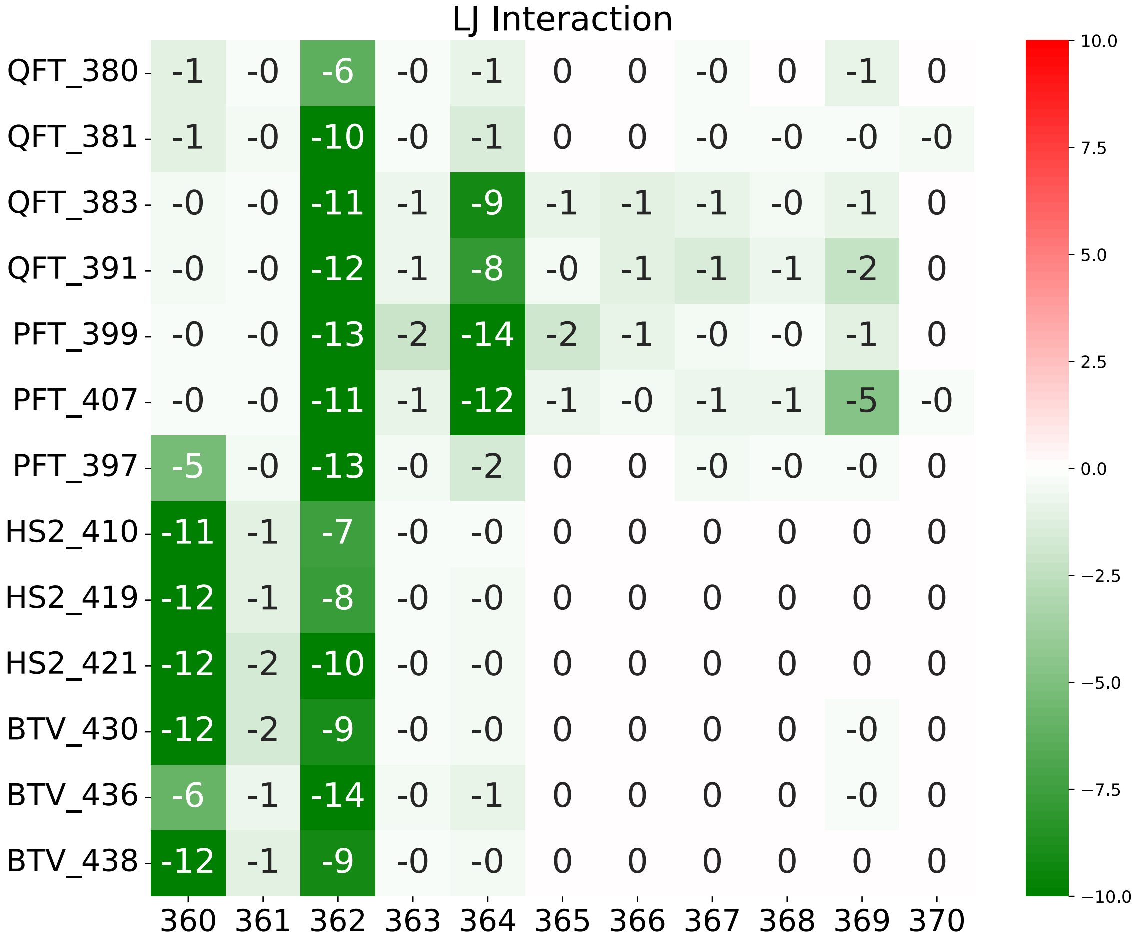

Figure S13: Lennard-Jones interaction energies (in kcal/mol) between tau peptide residues ILE360, THR361, HIS362, VAL363, PRO364, GLY365, GLY366, GLY367, ASN368, LYS369, LYS370, and the ligands pFTAA (PFT), qFTAA (QFT), HS276 (HS2), and bTVBT4 (BTV)

2.3 Smaller binding site model

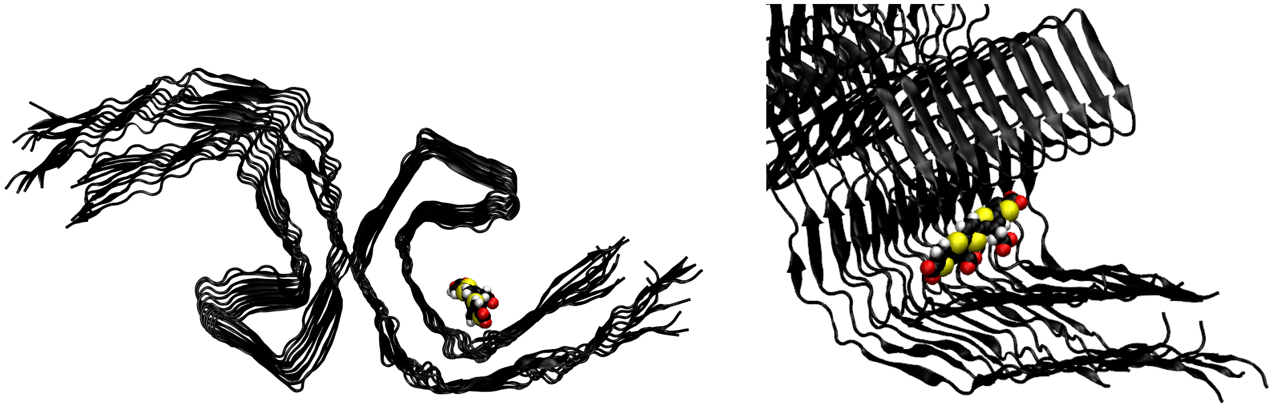

Figure S14: (a) Side view and (b) perspective view of the small binding site tau model derived from the half-pitch periodic tau model.

A small binding site model was created by cropping from a half-pitch model of an amyloid fibril to study the binding sites in greater detail. Each smaller binding site model consists of one ligand molecule and ten protofilaments of amyloid fibrils. Subsequently, water molecules and counter ions were added to a simulation box for each small binding site model, followed by energy minimization and equilibration processes. Following that, two independent 250 ns long (total accumulated time: 500 ns) MD simulations were performed in the NVT ensemble. To preserve the distance between adjacent protofilaments, a position restraint was applied to the outermost protofilaments of the amyloid fibrils.

In this study, we performed simulations for the interactions of the ligands pFTAA, qFTAA-CN, HS-276, and bTVBT-4 with amyloid beta fibrils, and of the ligands pFTAA, qFTAA-CN, and HS-276 with tau fibrils. For the interaction of bTVBT-4 with the tau small model, data was adopted from previous studies.[1] In the simulations of bTVBT-4 interacting with Aβ(1—42) fibril, we obtained a total accumulated simulation time of only 270 ns, as in both independent simulations the ligand detached from the binding site.

To analyze binding modes, we calculated planarity scores, explored shape space, and performed PMF calculations using simulation data from small models. The following subsections detail these analyses.

2.4 Planarity scores

To assess and compare the planarity of ligands in different environments, we calculated planarity scores derived from its molecular configuration. For each ligand, specific dihedral angles relevant to its structural planarity were identified and computed across all simulation frames. The planarity score for each frame was defined as the sum of the deviations of these dihedral angles from 90 degrees, using the following formula:

$$\text{Planarity Score} = \sum_i \frac{||\theta_i| - 90°|}{90°} \tag{1}$$

where θ_i represents the i-th dihedral angle. Next, we employed Gaussian Kernel Density Estimation (KDE) to generate probability density distributions of the planarity scores for each ligand within each environment. These normalized distributions were then plotted, allowing for a direct comparison of how the planarity of each ligand varies across different environmental.

Table S7: Planarity score and standard deviations for ligands in different environments

Ligand	Protein Environment		Water Environment
	Amyloid	tau	Planarity
pFTAA (4)	2.86 ± 0.31	2.86 ± 0.28	2.77 ± 0.31
qFTAA-CN (3)	2.30 ± 0.28	2.36 ± 0.24	2.15 ± 0.30
HS-276 (2)	1.37 ± 0.27	1.36 ± 0.27	1.26 ± 0.28
bTVBT4 (4)	3.35 ± 0.24	3.46 ± 0.22	3.38 ± 0.24
bTVBT4 (1)	0.81 ± 0.12	0.84 ± 0.11	0.82 ± 0.12

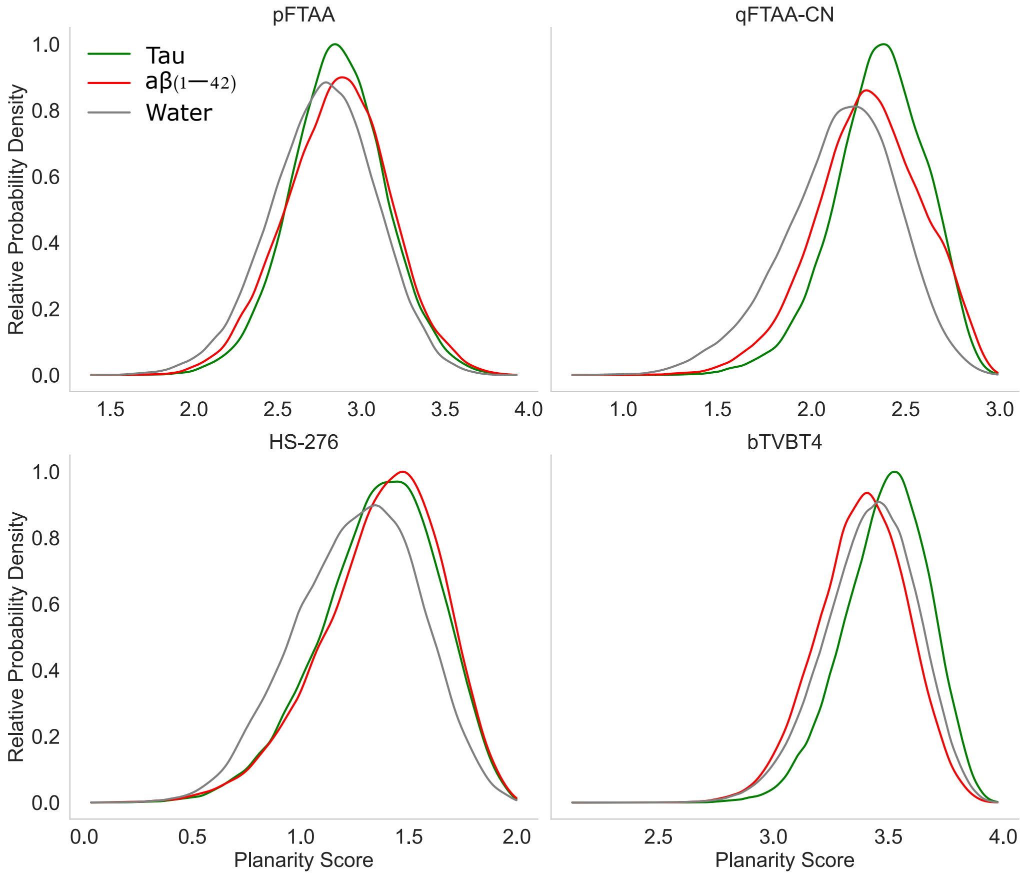

Figure S15: Relative probability density distributions of planarity scores for four ligands (pFTAA, qFTAA-CN, HS-276, and bTVBT4) across three environments: tau, amyloid-beta, and water.

2.5 Shape Space

The comparative analysis of ligand shape spaces in different environments was performed using ternary plots. These plots were based on the linear, planar, and isotropic data calculated by the VIAMD program for each conformation per simulated frame. For each ligand, the three coordinates (linear, planar, and isotropic) were normalized so that the contributions from each frame summed to one, allowing their representation within a ternary plot framework. The normalized data were then projected onto two-dimensional Cartesian coordinates using the Ternary Python library.[11] Subsequently, a two-dimensional Gaussian KDE was applied to the projected data to estimate the density distribution across the shape space. To highlight regions with significant conformational populations, a grid was established over the ternary plot, and the KDE was evaluated across this grid. Regions with densities below 5% of the maximum value were filtered out to exclude low-density areas. Finally, contour plots representing various density levels were created for each environment using custom colormaps that transition from white to a base color specific to each environment (e.g., grey for water, green for tau, red for Aβ(1–42)).

2.6 PMF calculation details

Using potential of mean force (PMF) calculations, the height of the atomistic free-energy barrier for ligand binding to amyloid fibrils at various key binding sites was determined. It was accomplished using an umbrella sampling technique and a weighted histogram analysis method. From the last frame obtained from an MD simulation of a small binding model of amyloid fibrils, the ligand was pulled away from a binding site until it no longer interacted with any amino acid residue of the fibril. The center of mass (COM) pulling was applied between amino acid residues at a binding site and a ligand. A pulling rate of 0.001 nm/ps and a 3000 kJ/mol spring constant were used. Using these pulled trajectories, snapshots were extracted and used as starting configurations for the umbrella sampling windows. Next, a short 100 ps NVT simulation for equilibration and a two ns NVT production simulation were

performed for each window. Using the weighted histogram analysis method (WHAM),[12,13] the results obtained from umbrella sampling were analyzed. The final binding profile was obtained by averaging two PMF profiles for each binding site.

References

(1) Todarwal, Y.; Gustafsson, C.; Thi Minh, N. N. et al. Tau Protein Binding Modes in Alzheimer's Disease for Cationic Luminescent Ligands. *The Journal of Physical Chemistry B* **2021**, *125*, 11628–11636.

(2) Berendsen, H.; van der Spoel, D.; van Drunen, R. GROMACS: A message-passing parallel molecular dynamics implementation. *Computer Physics Communications* **1995**, *91*, 43–56.

(3) Lindahl, E.; Hess, B.; van der Spoel, D. GROMACS 3.0: a package for molecular simulation and trajectory analysis. *Molecular modeling annual* **2001**, *7*, 306–317.

(4) Van Der Spoel, D.; Lindahl, E.; Hess, B. et al. GROMACS: Fast, flexible, and free. *Journal of Computational Chemistry* **2005**, *26*, 1701–1718.

(5) Hess, B.; Kutzner, C.; van der Spoel, D. et al. GROMACS 4: Algorithms for Highly Efficient, Load-Balanced, and Scalable Molecular Simulation. *Journal of Chemical Theory and Computation* **2008**, *4*, 435–447.

(6) Maier, J. A.; Martinez, C.; Kasavajhala, K. et al. ff14SB: Improving the Accuracy of Protein Side Chain and Backbone Parameters from ff99SB. *Journal of Chemical Theory and Computation* **2015**, *11*, 3696–3713.

(7) Jorgensen, W. L.; Chandrasekhar, J.; Madura, J. D. et al. Comparison of Simple Potential Functions for Simulating Liquid Water. *J. Chem. Phys.* **1983**, *79*, 926–935.

(8) Darden, T.; York, D.; Pedersen, L. Particle mesh Ewald: An N log(N) method for Ewald sums in large systems. *The Journal of Chemical Physics* **1993**, *98*, 10089–10092.

(9) Hess, B.; Bekker, H.; Berendsen, H. J. C. et al. LINCS: A linear constraint solver for molecular simulations. *Journal of Computational Chemistry* **1997**, *18*, 1463–1472.

(10) König, C.; Skånberg, R.; Hotz, I. et al. Binding sites for luminescent amyloid biomarkers from non-biased molecular dynamics simulations. *Chem. Commun.* **2018**, *54*, 3030–3033.

(11) et al, M. H. python-ternary: Ternary Plots in Python. *Zenodo 10.5281/zenodo.594435*

(12) Kumar, S.; Rosenberg, J. M.; Bouzida, D. et al. THE weighted histogram analysis method for free-energy calculations on biomolecules. I. The method. *Journal of Computational Chemistry* **1992**, *13*, 1011–1021.

(13) Hub, J. S.; de Groot, B. L.; van der Spoel, D. g_wham—A Free Weighted Histogram Analysis Implementation Including Robust Error and Autocorrelation Estimates. *Journal of Chemical Theory and Computation* **2010**, *6*, 3713–3720.
